# Efficacy and safety of N‐acetylcysteine in patients with vascular mild cognitive impairment (vMCI): Memory and antioxidant in vMCI Exercise Intervention Trial (MOVE‐IT)

**DOI:** 10.1002/alz70859_106789

**Published:** 2025-12-26

**Authors:** Yejin Kang, Nathan Herrmann, Damien Gallagher, Jinghan Jenny Chen, Ethan Mah, Kate Survilla, Danielle Vieira, Sandra E. Black, Ana C. Andreazza, Alex Kiss, Paul I. Oh, Joel Ramirez, Walter Swardfager, Susan Marzolini, Krista L Lanctôt

**Affiliations:** ^1^ University of Toronto, Toronto, ON Canada; ^2^ Sunnybrook Research Institute, Toronto, ON Canada; ^3^ Sunnybrook Health Sciences Centre, Toronto, ON Canada; ^4^ Center for Addiction and Mental Health, Toronto, ON Canada; ^5^ KITE Research Institute, University Health Network, Toronto, ON Canada; ^6^ Toronto Rehabilitation Institute, University Health Network, Toronto, ON Canada

## Abstract

**Background:**

Oxidative stress (OS) is implicated in the etiology of vascular mild cognitive impairment (vMCI), a form of cognitive decline originating from cerebrovascular pathology. vMCI is characterized by early deficits in executive function and memory, with OS suspected to contribute to its development. Although aerobic exercise has been shown to improve cognitive function and oxidative balance, it can also lead to an increase in OS. We hypothesized that the antioxidant N‐acetylcysteine (NAC) may help mitigate this damage by replenishing glutathione levels, particularly in those undergoing exercise interventions. This study evaluated the efficacy and safety of oral NAC for improving cognitive function in vMCI patients participating in a cardiac rehabilitation program.

**Method:**

In this 24‐week randomized clinical trial, patients with vMCI (1SD below the norm in executive function, memory, processing speed or working memory, with significant cardiovascular disease) were randomized to receive either NAC (2400mg/day) or placebo. Participants were recruited from the Toronto Rehab Institute‐Cardiac rehabilitation program. Cognitive function was assessed using a 60‐minute neuropsychological battery, with executive function as the primary outcome. Phonemic fluency, semantic fluency, and the Trail Making Test B were administered as part of the executive function. Z‐scores for each task were computed based on published age‐matched norms, and a composite z‐score was calculated for overall executive function. Data were analyzed using mixed‐effects models adjusting for relevant covariates. Adverse events (AEs) were monitored throughout the study period.

**Result:**

A total of 59 participants [mean(SD) age = 67.6(7.7), male(%) = 69.5%, mean(SD) MoCA= 22.5(3.2)] were randomized. Executive function improved over time (β=0.36, 95%CI=[0.20, 0.53], p<0.001), with no significant differences between NAC (n=29) and placebo (n=30) (β= ‐0.04, 95%CI= [‐0.28, 0.20], p=0.7) while controlling for sex, years of education, BMI, homocysteine levels, and VO2. NAC was well‐tolerated, with no significant differences in the frequency or severity of AEs between groups. Common AEs included dyspepsia, nausea, and headache. Most AEs resolved post‐treatment.

**Conclusion:**

NAC supplementation did not provide additional cognitive benefits beyond that of cardiac rehabilitation in patients with vMCI.

